# Tai Chi Training as a Primary Daily Care Plan for Better Balance Ability in People With Parkinson's Disease: An Opinion and Positioning Article

**DOI:** 10.3389/fneur.2021.812342

**Published:** 2021-12-24

**Authors:** Ting Zhang, Zhenyu Lv, Song Gao

**Affiliations:** ^1^College of Physical Education and Health Sciences, Zhejiang Normal University, Jinhua, China; ^2^University Hospital, Zhejiang Normal University, Jinhua, China; ^3^Department of Chinese Medicine, Naval Special Medical Center, Naval Medical University, Shanghai, China

**Keywords:** Parkinson's disease, fall, tai chi, balance ability, limitations

## Introduction

Parkinson's disease (PD) is a common degenerative disease of the central nervous system. Clinically, its incidence is second only to Alzheimer's disease, which seriously harms the health of middle-aged and elderly people (1). Main clinical manifestations of this disease include balance disorder, resting tremor, bradykinesia, and muscle stiffness, and this disease has a high incidence and disability rate. However, initial symptoms of PD are different, and the early symptoms are often ignored by people, which delays the optimal time to manage the disease. PD is closely related to age. Epidemiological surveys showed that the global prevalence of PD is 0.3%, among which the population over 65 years old accounts for 1–2%, and the prevalence rate over 85 years old increases to 3–5% (2, 3). A meta-analysis of people, both genders, with PD showed that men are at higher risk of PD than women (4). The progression of PD is unpredictable and may suddenly worsen. People with PD often complain that their symptoms clearly worsen within one year.

Fall is a balance disorder that often occurs in the late stage of PD. However, some studies have found that abnormal body swings occur in the early stages of PD, that is, mild balance dysfunction, which gradually worsens as the course of the disease progresses (5). Some researchers have found that people with Hoehn-Yahr stageII PD have balance adjustment disorders when they turn around (6). Newly diagnosed unmedicated people with PD have abnormal balance (7). Ultrasound of abnormal brain substantia nigra shows mild balance wobble in high-risk people with PD (8). Therefore, people with PD can have mild balance dysfunction in the early stage (9). As the course of the disease progresses, people with PD will inevitably show signs of abnormal dynamic balance, and even fall, leading to fractures and disability.

Although the apoptosis of dopaminergic cells in the substantia nigra striatum is the main cause of motor symptoms of PD, the balance disorder cannot be explained by lack of dopamine alone. Application of PET-CT found that apoptosis of the substantia nigra is closely related to motor retardation, but it has little to do with postural balance. It is well known that Medoba cannot alleviate or partially alleviate the symptoms of balance disorders in people with PD. Levodopa is the first-line drug for the treatment of PD. It has a good therapeutic effect on muscle stiffness and motor retardation, but there is no consensus on whether it can improve the symptoms of instability in PD (10, 11). In addition, long-term use of drugs can cause adverse drug reactions, such as nausea, vomiting, and orthostatic hypotension. Deep brain stimulation (DBS) is a surgical treatment for movement disorders such as PD (12). A study found that DBS stimulation of bilateral pontine nuclei can provide an effective treatment for alleviating gait and balance abnormalities in people with PD (13). In other studies, the effect of DBS on gait and balance disorders has been less successful and may even lead to freezing and increased gait imbalance (14). For the symptoms of PD, management rather than treatment is considered a more realistic strategy. Therefore, it is particularly important to actively seek safe and effective complementary and alternative therapies to improve the balance disorder in PD. Since 2002, Complementary and alternative therapy has been widely used in the United States and has attracted the attention of patients with neurological diseases (15). Short-term muscle stretching and functional electrical stimulation have also been shown to be effective in improving the gait of patients with PD (16, 17). Exercise therapy is considered to be an adjunct to medical and surgical treatments designed to maximize function, improve quality of life, and minimize or reduce complications (18). Although exercise therapy is recommended as an effective treatment for persons with PD, there is no uniform standard for specific exercise patterns (19, 20).

Fear of falling, decreased muscle strength, and decreased proprioception are the main factors that cause falls. A study reported that the elderly showed lower muscle use efficiency and greater postural swings in standing balance tasks, which means that the elderly has a greater risk of falls (21). Lelard et al. (22) considered that any form of physical activities can increase confidence in maintaining balance, and that strength and proprioception training is the most suitable balance exercises for healthy elderly people. However, due to practical considerations, the elderly, especially elderly people with PD, have a low chance of participating in special strength training.

Tai chi, a popular traditional Chinese martial art, has gradually developed into an exercise therapy. As we all know, tai chi is a form of exercise that requires long-term practice and continuous improvement, and Yang tai chi is the most popular (23). Tai chi can increase muscle strength and improve body coordination. Unlike other complementary and alternative therapies, tai chi training is very economical, and there is no need to consider whether the state can provide subsidies. According to a report, each tai chi class in the United States is worth USD 3.5, which is acceptable to most practitioners (24). Practitioners can choose actions that improve balance and flexibility rather than the entire set of actions. In recent years, tai chi has been proven to improve many diseases, such as chronic obstructive pulmonary disease, heart failure, and knee osteoarthritis (25). In the tai chi training process, participants continuously adjust their center of gravity while moving slowly in multiple directions to maintain their body balance. A meta-analysis showed that tai chi has a promoting effect on balance, and it is believed that it has a good effect on the strengthening of proprioception (26, 27). A study by Guo et al. (28) found that compared with a control group who had not practiced tai chi, the elderly who had practiced tai chi for an average of 9 years or more had a great advantage in proprioception of the knee and ankle joints. This shows that long-term tai chi exercise helps maintain or strengthen the proprioception of the elderly.

The balance and stability of people with PD are seriously threatened, and tai chi training has potential healing effects. Therefore, this article aims to outline the key role of tai chi training in enhancing balance ability and preventing falls in people with PD. Moreover, the authors emphasize the limitations and challenges of this research on tai chi to improve balance ability in PD.

## Tai Chi Promotes the Balance Ability of People With Pd

Human daily activities cannot be performed without balance ability. Studies have shown that elderly people with PD are more likely to be admitted to a hospital for fractures due to falls (29). Therefore, seeking active and effective exercise methods to enhance the balance ability of elderly people with PD is particularly important for improving their quality of life. Tai chi has slow rhythm and continuous movements. It enhances the balance ability of people with PD by constantly shifting the center of gravity. The decreased balance ability of people with PD is closely related to loss of posture control. Tai chi training finely controls joints through muscle coordination. In addition, tai chi training can strengthen the sensory stimulation of the limbs and lower limb muscles, which is very important for the improvement of the movement and balance ability of people with PD.

In order to investigate how tai chi reduces the risk of falls, Rahal et al. (30) compared the balance ability between healthy elderly people who practiced Tai Chi and ballroom dancers. They found that the tai chi group had faster walking speed and shorter transfer time, and that in the sit-to-stand test the tai chi group had better balance performance in the final standing posture. Besides, Zhou et al. (31) reported the influence of tai chi training on posture control of elderly women. When the tai chi group shifted the center of gravity as quickly as possible to complete the special orientation posture, and remain stable without falling, the overall, lateral, and anteroposterior diameter swing paths of the center of gravity were smaller than those of the control group. The study by Holmes et al. (32) found that tai chi can reduce the swing of the body's center of gravity caused by respiratory disturbance, thereby reducing the instability of the body. Zhou et al. (33) found that Tai Chi can effectively increase muscle strength of the lower limbs for elderly people. Furthermore, crossing obstacles is a behavior that occurs in daily life, which requires better dynamic balance ability. Chang et al. (34) reported that when crossing obstacles, the flexion angle of the hip joint in a tai chi group was significantly greater when raising the leg, and that the tai chi group had a larger stride, faster stepping speed, and shorter time required to cross an obstacle. Therefore, tai chi training has a positive effect on maintaining balance when crossing obstacles. Another research by Zou et al. (35) showed that both the 24-style tai chi and the modified Chen-style tai chi can effectively enhance the balance function and adaptability of body posture control in elderly people with PD. Recent studies have shown that tai chi exercise can better promote balance function, and that it is significantly better than stretching exercises and multi-modal exercises in reducing the incidence of moderate injury falls and severe injury falls (36, 37). However, the results of this study cannot determine whether Tai Chi is suitable for elderly PD patients since the participants were not PD patients though over 65 years old.

Tai Chi can effectively improve the static balance ability of middle-aged and elderly people, but there is no direct evidence to improve PD patients' static balance ability (38). In tai chi movements, such as “brushing keen and twisting step” and “parting the wild horse's mane,” the legs should be open to a larger and suitable angle, and the maximum swing should be emphasized when doing the movements (39, 40). The knees should be bent and squat with the knee joints on the frontal axis. The angle between the sagittal axis and the sagittal axis is increased, and this posture can improve the control ability of the practitioner's lower limbs. This may be the key reason for long-term tai chi training to improve posture control ability and reduce the ellipse area that represents the static balance ability.

The significance of tai chi for the treatment of PD is mainly to improve gait and strengthen neuromuscular control to reduce the risk of falls. Characteristics of the included studies are shown in Table 1. A research study by Li et al. (39) found that tai chi can maintain and improve various body functions of elderly people with PD and that it is a very effective exercise therapy. In its follow-up study (41), 195 patients with PD, Hoehn-Yahr stages 1 to 4, were randomly divided into tai chi group, resistance training group, and stretching training group, each for 60 min/time, 2 times/week, for a total of 24 weeks of intervention. The results showed that the tai chi group performed better than the resistance group in terms of maximum excursion, with a difference of 5.55 percentage points between the groups (95% confidence interval [CI], 1.12–9.97, *P* = 0.01). In terms of directional control, the difference between groups was 10.45 percentage points (95% CI, 3.89–17, *P* = 0.002). Compared with the stretching group, the tai chi group had more obvious differences between the groups in maximum excursion and direction control. This showed that tai chi has significant effects on improving postural stability and functional ability of people with mild to moderate PD. Liu (42) et al. showed that tai chi exercise-assisted balance and gait training can reduce the occurrence of falls in people with PD, and that the improvement of traction in the tai chi training group was significantly better than that of the control group. A study by Gao et al. (43) found that the 12-week Yang Style tai chi was better than the control group in improving the Berg balance scale (*p* < 0.05), and that the number of falls in the Tai Chi group during the 6-month follow-up period was significantly less than that in the control group. The number of falls in the tai chi group was 0.3 ± 0.62 times, and in the control group it was = 0.64 ± 0.74 times (*p* < 0.05). In a preliminary experiment conducted by Hackney et al. (44), 30 people with PD were randomly divided into a tai chi group and a control group. The tai chi group intervened for 1 h a day twice a week, for a total of 10 weeks. The control group did not intervene. The results showed that before and after exercise, the tai chi group had significant changes in the Berg Balance Scale, Unified Parkinson's Disease Rating Scale (UPDRS), Timed Up and Go, tandem stance test, 6-minute walk, and backward walking compared with the control group.

**Table 1:** Characteristics of the included studies.

**Study**	**Participants**	**Interventions**	**Outcomes**
Li et al. (39)	*n* = 17 Age: 71.51 (± 5.4) y HY scale1–3 stage ability to walk with/without aids	Yang style Tai Chi-based stepping exercises vs. no intervention 90min, 5 times/week, 5 weeks	50-ft walk, TUGT, FRT
Li et al. (41)	*n* = 195 Age: 40–85y HY scale scale1–4 stage ability to walk with/without aids	Tai Chi vs. resistance training vs. stretching 60 min, 2 times/week, 24 weeks	Falls, TUG, UPDRS-III, limit-of stability, FRT, gait, strength
Gao et al. (43)	*n* = 76 Age>40 y Independent walking≥1 fall during past 1 y	Yang style Tai Chi vs. no intervention 60 min, 3 times/week, 12 weeks	Occurrences of falls, BBS, TUGT, UPDRS-III
Amano et al. (47)	*n* = 45 Age: 50–7 0y HY scale 2–3 stage ability to walk with/without aids	Tai Chi vs. Qigong vs. no intervention 60 min, 2 times/week, 16 weeks	GI, gait, UPDRS-III
Hackney et al. (44)	*n* = 26 Age > 40 y HY scale 1.5–3 stageindependent walking with/without aids for 3 m	Yang short style Tai Chi vs. no intervention 60 min, 2 times/week, 20 sessions	BBS, TUG, TS, UPDRS-III,OLS, GAITRite, 6MWT

*HY scale, Hoehn and Yahr scale; TUGT, timed up and go test; FRT, functional reach test; UPDRS, Unified Parkinson's Disease Rating Scale; BBS, Berg Balance Scale; GI, gait initiation; TS, tandem stance; OLS, one-leg stance; 6MWT, 6-min walk test*.

However, there are only few studies that believe tai chi has no significant effect on reducing falls in people with PD (45, 46). Amano et al. (47) found that tai chi training had no effect on the gait and posture control of people with PD, and that no improvement in people's balance ability was observed. It was speculated that the reason could be the short duration of training, which was only 16 weeks and only 2 to 3 practice sessions per week.

## Limitations of Current Tai Chi Research

There are some limitations in the research on Tai Chi improving the balance ability of people with PD. This may be attributed to the following reasons: at present, most clinical control studies recruit subjects who have no foundation in tai chi training. After randomization, the trial group will be given tai chi training and practice for not more than 6 months to evaluate the difference with the control group (46, 47). Therefore, the reason for the diverse conclusions is the difference in the standard degree of tai chi movement and length of training. Moreover, when formulating tai chi to improve the balance ability of people with PD, the characteristics of different tai chi categories should be considered, and unified Tai Chi training movements should be performed. The selection and modification of Tai Chi actions for PD needs to focus on the severity of the disease of people with PD to enhance the reproducibility and generalization of the research results.

Tai Chi, as a medium-intensity aerobic exercise, with frequency of training of three times a week, can meet the recommended standards of American College of Sports Medicine (ACSM). Furthermore, tai chi is a kind of physical and mental exercise. During exercise, breathing and soothing music should be used to effectively improve the mood of people with PD, and it is beneficial to overcome the fear of falling. Compared with the rehabilitation training content of modern medicine, tai chi training improves the abnormal gait and balance disorders of elderly people with PD. It does not require special equipment and venues, and the exercise intensity and difficulty are not challenging, which is convenient for people with PD to practice. Tai chi improves the movement and balance abilities of people with PD, effectively reducing physical and psychological burdens of caregivers, and it has high social and economic benefits.

## Conclusion

The authors of this article point out that tai chi can improve balance ability and reduce the risk of falls in people with mild to moderate PD. It is an effective non-drug intervention. In addition, in view of the differences in research results, the existing problems of Tai Chi intervention in PD should be deeply analyzed. This is also a key issue that needs attention and consideration in the future research. However, more evidence-based research is needed to prove the effectiveness of tai chi in improving balance ability and preventing falls of people with PD. Researchers designing tai chi movements should take into full consideration the special physical conditions of people with PD, movements that are simple and easy to learn while having good effects on improving balance ability. Based on a good mass foundation, tai chi movement will be more widely used and promoted as a daily care plan for people with PD.

## Author Contributions

TZ and ZL conceived the manuscript and revised the drafts. SG wrote the first draft. All authors contributed to the article and approved the submitted version.

## Funding

This study was supported by the College of Physical Education and Health Sciences, Zhejiang Normal University, Jinhua, China.

## Conflict of Interest

The authors declare that the research was conducted in the absence of any commercial or financial relationships that could be construed as a potential conflict of interest.

## Publisher's Note

All claims expressed in this article are solely those of the authors and do not necessarily represent those of their affiliated organizations, or those of the publisher, the editors and the reviewers. Any product that may be evaluated in this article, or claim that may be made by its manufacturer, is not guaranteed or endorsed by the publisher.

## References

[1] Arii Y Sawada Y Kawamura K Miyake S Taichi Y Izumi Y. Immediate effect of spinal magnetic stimulation on camptocormia in Parkinson's disease. J Neurol Neurosurg Psychiatry. (2014) 85:1221–6. 10.1136/jnnp-2014-307651 24780955

[2] de Lau LML Breteler MMB. Epidemiology of Parkinson's disease. Lancet Neurol. (2006) 5:525–35 10.1016/S1474-4422(06)70471-9 16713924

[3] Elbaz A Carcaillon L Kab S Moisan F. Epidemiology of Parkinson's disease. Rev Neurol (Paris). (2016) 172:14–26. 10.1016/j.neurol.2015.09.012 26718594

[4] Taylor KSM Cook JA Counsell CE. Heterogeneity in male to female risk for Parkinson's disease. J Neurol Neurosurg Psychiatry. (2007) 78:905–6 10.1136/jnnp.2006.104695 17635983 PMC2117744

[5] Kim SD Allen NE Canning CG Fung VSC. Postural instability in patients with Parkinson's disease. epidemiology, pathophysiology and management. CNS Drugs. (2013) 27:97–112. 10.1007/s40263-012-0012-3 23076544

[6] Song J Sigward S Fisher B Salem GJ. Altered dynamic postural control during step turning in persons with early-stage parkinson's disease. Parkinsons Dis. (2012) 2012:386962. 10.1155/2012/386962 22518349 PMC3306994

[7] Mancini M Horak FB Zampieri C Carlson-Kuhta P Nutt JG Chiari L. Trunk accelerometry reveals postural instability in untreated Parkinson's disease. Parkinsonism Relat Disord. (2011) 17:557–62. 10.1016/j.parkreldis.2011.05.010 21641263 PMC5327861

[8] Maetzler W Mancini M Liepelt-Scarfone I Müller K Becker C van Lummel RC. Impaired trunk stability in individuals at high risk for Parkinson's disease. PLoS ONE. (2012) 7:e32240. 10.1371/journal.pone.0032240 22457713 PMC3311622

[9] Delafontaine A Hansen C Marolleau I Kratzenstein S Gouelle A. Effect of a concurrent cognitive task, with stabilizing visual information and withdrawal, on body sway adaptation of Parkinsonian's patients in an off-medication state: a controlled study. Sensors (Basel). (2020) 20:18. 10.3390/s20185059 32899926 PMC7571225

[10] Nova IC Perracini MR Ferraz HB. Levodopa effect upon functional balance of Parkinson's disease patients. Parkinsonism Relat Disord. (2004) 10:411–5 10.1016/j.parkreldis.2004.04.004 15465397

[11] Rocchi L Chiari L Horak FB. Effects of deep brain stimulation and levodopa on postural sway in Parkinson's disease. J Neurol Neurosurg Psychiatry. (2002) 73:267–74 10.1136/jnnp.73.3.267 12185157 PMC1738049

[12] Fukaya C Yamamoto T. Deep brain stimulation for Parkinson's disease: recent trends and future direction. Neurol Med Chir. (2015) 55:422–31. 10.2176/nmc.ra.2014-0446 25925761 PMC4628170

[13] Stefani A Lozano AM Peppe A Stanzione P Galati S Tropepi D. Bilateral deep brain stimulation of the pedunculopontine and subthalamic nuclei in severe Parkinson's disease. Brain. (2007) 130:1596–607 10.1093/brain/awl346 17251240

[14] Collomb-Clerc A Welter ML. Effects of deep brain stimulation on balance and gait in patients with Parkinson's disease: A systematic neurophysiological review. Neurophysiol Clin. (2015) 45:371–88. 10.1016/j.neucli.2015.07.001 26319759

[15] Du S Dong J Zhang H Jin S Xu G Liu Z. Taichi exercise for self-rated sleep quality in older people: a systematic review and meta-analysis. Int J Nurs Stud. (2015) 52:368–79. 10.1016/j.ijnurstu.2014.05.009 24934815

[16] Delafontaine A Fourcade P Zemouri A Diakhaté DG Saiydoun G Yiou E. In patients with parkinson's disease in an off-medication state, does bilateral electrostimulation of tibialis anterior improve anticipatory postural adjustments during gait initiation? Front Hum Neurosci. (2021) 15:692651. 10.3389/fnhum.2021.692651 34366815 PMC8337069

[17] Vialleron T Delafontaine A Millerioux I Memari S Fourcade P Yiou E. Acute effects of short-term stretching of the triceps surae on ankle mobility and gait initiation in patients with Parkinson's disease. Clin Biomech. (2021) 89:105449. 10.1016/j.clinbiomech.2021.105449 34418858

[18] Abbruzzese G Marchese R Avanzino L Pelosin E. Rehabilitation for Parkinson's disease: Current outlook and future challenges. Parkinsonism Relat Disord. (2016) 22 Suppl 1:S60–S4. 10.1016/j.parkreldis.2015.09.005 26360239

[19] Luan X Tian X Zhang H Huang R Li N Chen P. Exercise as a prescription for patients with various diseases. J Sport Health Sci. (2019) 8:422–1. 10.1016/j.jshs.2019.04.002 31534817 PMC6742679

[20] Guo S Huang Y Zhang Y Huang H Hong S Liu T. Impacts of exercise interventions on different diseases and organ functions in mice. J Sport Health Sci. (2020) 9:53–73. 10.1016/j.jshs.2019.07.004 31921481 PMC6943779

[21] Donath L Kurz E Roth R Zahner L Faude O. Leg and trunk muscle coordination and postural sway during increasingly difficult standing balance tasks in young and older adults. Maturitas. (2016) 91:60–8. 10.1016/j.maturitas.2016.05.010 27451322

[22] Lelard T Ahmaidi S. Effects of physical training on age-related balance and postural control. Neurophysiol Clin. (2015) 45:357–69. 10.1016/j.neucli.2015.09.008 26548366

[23] Huang Z-G Feng Y-H Li Y-H Lv C-S. Systematic review and meta-analysis: Tai Chi for preventing falls in older adults. BMJ Open. (2017) 7:e013661. 10.1136/bmjopen-2016-013661 28167744 PMC5293999

[24] Li F Harmer P McAuley E Duncan TE Duncan SC Chaumeton N. An evaluation of the effects of Tai Chi exercise on physical function among older persons: a randomized contolled trial. Ann Behav Med. (2001) 23:139–46 10.1207/S15324796ABM2302_9 11394556

[25] Lan C Lai J-S Chen S-Y. Tai Chi Chuan: an ancient wisdom on exercise and health promotion. Sports Med. (2002) 32:217–24 10.2165/00007256-200232040-00001 11929351

[26] Winser SJ Tsang WW Krishnamurthy K Kannan P. Does Tai Chi improve balance and reduce falls incidence in neurological disorders? a systematic review and meta-analysis. Clin Rehabil. (2018) 32:1157–68. 10.1177/0269215518773442 29737198

[27] Zou L Han J Li C Yeung AS Hui SS-C Tsang WWN. Effects of tai chi on lower limb proprioception in adults aged over 55: a systematic review and meta-analysis. Arch Phys Med Rehabil. (2019) 100:1102–13. 10.1016/j.apmr.2018.07.425 30125554

[28] Guo L-y Yang C-p You Y-l Chen S-k Yang C-h Hou Y-y. Underlying mechanisms of Tai-Chi-Chuan training for improving balance ability in the elders. Chin J Integr Med. (2014) 20:409–15. 10.1007/s11655-013-1533-4 24952168

[29] Lan C Chen S-Y Lai J-S Wong AM-K. Tai chi chuan in medicine and health promotion. Evid Based Complement Alternat Med. (2013) 2013:502131. 10.1155/2013/502131 24159346 PMC3789446

[30] Rahal MA Alonso AC Andrusaitis FR Rodrigues TS Speciali DS Greve JMDA. Analysis of static and dynamic balance in healthy elderly practitioners of Tai Chi Chuan versus ballroom dancing. Clinics (São Paulo). (2015) 70:157–61. 10.6061/clinics/2015(03)01 26017644 PMC4449465

[31] Zhou J Chang S Cong Y Qin M Sun W Lian J. Effects of 24 weeks of Tai Chi Exercise on postural control among elderly women. Res Sports Med. (2015) 23:302–14. 10.1080/15438627.2015.1040918 26223978

[32] Holmes ML Manor B Hsieh W-h Hu K Lipsitz LA Li L. Tai Chi training reduced coupling between respiration and postural control. Neurosci Lett. (2016) 610:60–5. 10.1016/j.neulet.2015.10.053 26518241 PMC4695235

[33] Zhou M Peng N Dai Q Li H-W Shi R-G Huang W. Effect of Tai Chi on muscle strength of the lower extremities in the elderly. Chin J Integr Med. (2016) 22:861–6 10.1007/s11655-015-2104-7 26015074

[34] Chang Y-T Huang C-F Chang J-H. The effect of tai chi chuan on obstacle crossing strategy in older adults. Res Sports Med. (2015) 23:315–29. 10.1080/15438627.2015.1040920 26114218

[35] Zou L Loprinzi PD Yu JJ Yang L Li C Yeung AS. Superior effects of modified chen-style tai chi versus 24-style tai chi on cognitive function, fitness, and balance performance in adults over 55. Brain Sci. (2019) 9:5. 10.3390/brainsci9050102 31060221 PMC6562620

[36] Moreland B Kakara R Henry A. Trends in nonfatal falls and fall-related injuries among adults aged ≥65 Years - United States, 2012-2018. MMWR Morb Mortal Wkly Rep. (2020) 69:875–81. 10.15585/mmwr.mm6927a5 32644982 PMC7732363

[37] Li F Harmer P Eckstrom E Fitzgerald K Chou L-S Liu Y. Effectiveness of tai ji quan vs multimodal and stretching exercise interventions for reducing injurious falls in older adults at high risk of falling: follow-up analysis of a randomized clinical trial. JAMA Netw Open. (2019) 2:e188280. 10.1001/jamanetworkopen.2018.8280 30768195 PMC6484587

[38] Li Y Devault CN Van Oteghen S. Effects of extended Tai Chi intervention on balance and selected motor functions of the elderly. Am J Chin Med. (2007) 35:383–91 10.1142/S0192415X07004904 17597497

[39] Li F Harmer P Fisher KJ Xu J Fitzgerald K Vongjaturapat N. Tai Chi-based exercise for older adults with Parkinson's disease: a pilot-program evaluation. J Aging Phys Act. (2007) 15:139–51 10.1123/japa.15.2.139 17556781

[40] Schleicher MM Wedam L Wu G. Review of Tai Chi as an effective exercise on falls prevention in elderly. Res Sports Med. (2012) 20:37–58. 10.1080/15438627.2012.634697 22242736

[41] Li F Harmer P Fitzgerald K Eckstrom E Stock R Galver J. Tai chi and postural stability in patients with Parkinson's disease. N Engl J Med. (2012) 366:511–9. 10.1056/NEJMoa1107911 22316445 PMC3285459

[42] Liu H-H Yeh N-C Wu Y-F Yang Y-R Wang R-Y Cheng F-Y. Effects of Tai Chi exercise on reducing falls and improving balance performance in parkinson's disease: a meta-analysis. Parkinsons Dis. (2019) 2019:9626934. 10.1155/2019/9626934 30918623 PMC6409066

[43] Gao Q Leung A Yang Y Wei Q Guan M Jia C. Effects of Tai Chi on balance and fall prevention in Parkinson's disease: a randomized controlled trial. Clin Rehabil. (2014) 28:748–53 10.1177/0269215514521044 24519923

[44] Hackney ME Earhart GM. Tai Chi improves balance and mobility in people with Parkinson disease. Gait Posture. (2008) 28:456–60. 10.1016/j.gaitpost.2008.02.005 18378456 PMC2552999

[45] Taylor-Piliae RE Hoke TM Hepworth JT Latt LD Najafi B Coull BM. Effect of Tai Chi on physical function, fall rates and quality of life among older stroke survivors. Arch Phys Med Rehabil. (2014) 95:816–24. 10.1016/j.apmr.2014.01.001 24440643

[46] Kurlan R Evans R Wrigley S McPartland S Bustami R Cotter A. Tai Chi in Parkinson!s Disease: a preliminary randomized, controlled, and rater-blinded study. Adv Parkinson's Dis. (2015) :9–12. 10.4236/apd.2015.41002

[47] Amano S Nocera JR Vallabhajosula S Juncos JL Gregor RJ Waddell DE. The effect of Tai Chi exercise on gait initiation and gait performance in persons with Parkinson's disease. Parkinsonism Relat Disord. (2013) 19:955–60. 10.1016/j.parkreldis.2013.06.007 23835431 PMC3825828

